# Serum Levels of Caspase-Cleaved Cytokeratin-18 in Patients with Severe Traumatic Brain Injury Are Associated with Mortality: A Pilot Study

**DOI:** 10.1371/journal.pone.0121739

**Published:** 2015-03-30

**Authors:** Leonardo Lorente, María M. Martín, Agustín F. González-Rivero, Mónica Argueso, Luis Ramos, Jordi Solé-Violán, Juan J. Cáceres, Alejandro Jiménez, Juan M. Borreguero-León

**Affiliations:** 1 Intensive Care Unit, Hospital Universitario de Canarias, La Laguna, Tenerife, Spain; 2 Intensive Care Unit, Hospital Universitario Nuestra Señora de Candelaria, Santa Cruz de Tenerife, Spain; 3 Laboratory Deparment, Hospital Universitario de Canarias, Laguna, Tenerife, Spain; 4 Intensive Care Unit, Hospital Clínico Universitario de Valencia, Valencia, Spain; 5 Intensive Care Unit, Hospital General de La Palma, Breña Alta, La Palma, Spain; 6 Intensive Care Unit, Hospital Universitario Dr. Negrín, CIBERES, Las Palmas de Gran Canaria, Spain; 7 Intensive Care Unit, Hospital Insular, Las Palmas de Gran Canaria, Spain; 8 Research Unit, Hospital Universitario de Canarias, La Laguna, Tenerife, Spain; Fraunhofer Institute for Cell Therapy and Immunology, GERMANY

## Abstract

**Objective:**

There have been found apoptotic changes in brain tissue samples from animals and humans after a traumatic brain injury (TBI). The protein cytokeratin 18 (CK-18), present in epithelial cells, is cleaved by the action of caspases during apoptosis, and the resulting fragments are released into the blood as caspase-cleaved CK (CCCK)-18. Circulating levels of CCCK-18, as biomarker of apoptosis, have been determined in patients with different processes; however, it has not been explored in TBI patients. Thus, the objective of this study was to determine whether there is an association between serum CCCK-18 levels and mortality and whether such levels could be used as a biomarker to predict outcomes in TBI patients.

**Methods:**

A prospective, observational, multicenter study carried out in six Spanish Intensive Care Units. We included patients with severe TBI defined as Glasgow Coma Scale (GCS) lower than 9; and were excluded those patients with Injury Severity Score (ISS) in non-cranial aspects higher than 9. We measured serum CCCK-18 levels at admission. The end-point of the study was 30-day mortality.

**Results:**

Surviving patients (n = 73) showed lower serum CCCK-18 levels (P = 0.003) than non-survivors (n = 27). On ROC analysis, the area under the curve (AUC) for serum CCCK-18 levels as predictor of 30-day mortality was 0.69 (95% CI = 0.59–0.78; P = 0.006). We found in survival analysis that patients with serum CCCK-18 higher than 201 u/L had higher 30-day mortality than patients with lower levels (Hazard ratio = 3.9; 95% CI = 1.81–8.34; P<0.001). Regression analyses showed that serum CCCK-18 levels higher than 201 u/L were associated with 30-day mortality (OR = 8.476; 95% CI = 2.087–34.434; P = 0.003) after controlling for age and GCS.

**Conclusions:**

The novel finding of our study was that serum CCCK-18 levels are associated with 30-day mortality and could be used as a prognostic biomarker in patients with severe TBI.

## Introduction

Traumatic brain injury (TBI) is an important cause of death, disability, and health resource consumption [1]. Head trauma causes two types of injury in the neural tissue. One is the primary injury, which refers to the initial physical forces applied to the brain at the moment of the impact. The other is the secondary injury, which develops over a period of hours or days later, involving neuroinflammatory response, free radical generation and apoptosis.

The apoptotic process is one in which cells are actively eliminated via a programmed pathway during morphogenesis, tissue remodeling, and resolution of the immune response [2]. There have been found apoptotic changes in brain tissue samples from animals [3–5] and humans [6,7] after a traumatic brain injury (TBI). In addition, TBI may cause an systemic inflammatory response syndrome (SIRS) [8] and SIRS could activate cellular apoptosis [9].

Cytokeratins (CK) are proteins of intermediate filaments found in the intracytoplasmic cytoskeleton of epithelial tissue. The molecular weight of which ranges from 40 to 68 kDa. Until now there are 20 distinct CKs named as CK-1 to CK-20. In the cytoplasm, the CK filaments conform a complex network which extends from the surface of the nucleus to the cell membrane. CK filaments have important implications in static functions of cells (providing tensile strength to the cells) and in dynamic cellular processes (such as mitosis, cell movement and differentiation) [10]. CK-18 is cleaved at various sites by the action of caspases during apoptosis, and the resulting fragments are released into the blood [11]. Caspase-cleaved CK (CCCK)-18 can be determined using a monoclonal antibody (M30) [12,13].

Circulating levels of CCCK-18, as biomarker of apoptosis, have been studied in patients with liver [14–17], tumoral [18,19], graft-versus-host [20] and septic processes [21–23]. However, they have not been explored in TBI patients. Thus, the aim of this study was to determine whether there is an association between serum CCCK-18 levels and mortality and whether such levels could be used as a biomarker to predict outcomes in TBI patients.

## Methods

### Design and Subjects

A prospective, observational, multicenter study carried out in six Spanish Intensive Care Units between 2009–2012. The study was approved by the Institutional Review Board of the 6 participating hospitals: Hospital Universitario de Canarias (La Laguna), Hospital Universitario Nuestra Señora de Candelaria (Santa Cruz de Tenerife), Hospital Clínico Universitario de Valencia (Valencia), Hospital General de La Palma (La Palma), Hospital Universitario Dr. Negrín (Las Palmas de Gran Canaria), Hospital Insular (Las Palmas de Gran Canaria). Written informed consent from the patients or from their legal guardians was obtained.

The study included 100 patients with severe TBI. The same patient cohort was described in detail in a previously published study by our team [24]. The blood samples were frozen until determination of serum CCCK-18 levels. Our current study contributes to the base of knowledge compared to our prior work [24] in that apoptosis in blood has not been explored in TBI patients; and from a therapeutic perspective, modulators of apoptotic activity could be used as a new class of drugs for the treatment of TBI.

We used Glasgow Coma Scale (GCS) [25] to determine TBI severity, and severe TBI was defined as GCS lower than 9 points.

Patients with Injury Severity Score (ISS) [26] in non-cranial aspects higher than 9 points, age less than 18 years, pregnancy, inflammatory or malignant disease were excluded of the study.

### Variables recorded

We recorded the following variables for each patient: age, Acute Physiology and Chronic Health Evaluation II (APACHE II) score [27], activated partial thromboplastin time (aPTT), bilirubin, CCCK-18, cerebral perfusion pressure (CPP), creatinine, fibrinogen, GCS, glycemia, hemoglobin, ICP, international normalized ratio (INR), ISS, lactic acid, leukocytes, pressure of arterial oxygen (PaO2), fraction inspired oxygen (FI0_2_), platelets, sodium, temperature, gender, and brain lesion according to Marshall computer tomography (CT) classification [28]. Marshall CT lesion classification [28] is as follows: Class I or diffuse injury I when there is not visible pathology. Class II or diffuse injury II when cisterns are present with midline shift 0–5 mm, and there is not high- or mixed-density lesion > 25 cc. Class III or diffuse injury III (swelling) when cisterns are compressed or absent with midline shift 0–5 mm, and there is not high- or mixed-density lesion > 25 cc. Class IV or diffuse injury IV (shift) when there is a midline shift > 5 mm, and there is not high- or mixed-density lesion > 25 cc. Class V or Evacuated mass lesion when any lesion was evacuated. Class V or non-evacuated mass lesion when there is a high- or mixed-density lesion > 25 cc not surgically evacuated. The endpoint of the study was 30-day mortality

### Serum CCCK-18 analysis

Blood samples were collected in tubes with separator gel to obtain serum from 100 patients with severe TBI at admission to measure concentrations of CCCK-18. After coagulation during 10 min at room temperature, serum was obtained by centrifugation at 1000g for 15 min. The samples were aliquoted and frozen at -80°C until determination. All determinations were performed by laboratory technicians blinded to all clinical data. Assays were performed at the Laboratory Department of the Hospital Universitario de Canarias (La Laguna, Tenerife, Spain). We determine serum CCCK-18 levels by enzyme-linked immunosorbent assay (ELISA) using M30 Apoptosense ELISA (PEVIVA AB, Bromma, Sweden), lot PE-0133. The intra- and inter-assay CV were <10%. The detection limit for the assay was 25 U/L.

### Statistical Methods

Quantitative variables are reported as medians and interquartile ranges, and were compared with Wilcoxon-Mann-Whitney test. Qualitative variables are reported as frequencies and percentages and were compared with Chi-squared test.

We used a receiver operating characteristic (ROC) analysis to determine the goodness-of-fit of serum CCCK-18 levels to predict 30-day mortality. We carried out a Kaplan-Meier analysis to compare 30-day survival according to serum CCCK-18 levels lower/higher than 201u/L. We used dot-plot to represent serum CCCK-18 levels in 30-day surviving and non-surviving patients.

We carried out a multiple binomial logistic regression analysis to predict 30-day mortality. We constructed two multiple binomial logistic regression models with only three predictor variables in each model to avoid an over fitting effect since the number of events (death) was 27. We included serum CCCK-18 levels, age and GCS in the first model; and serum CCCK-18 levels, CT classification and APACHE-II score in the second model. Before including the variable CT classification in the regression analysis, it was recoded according to the risk of death observed in the bivariate analysis as high risk (CT types 3, 4 and 6) and low risk (CT types 2 and 5) since we found that 5 of 18 (27.8%) patients with CT classification type 3 died during the first 30 days, 6 of 16 (37.5%) with type 4, 8 of 11 (72.7%) with type 6, 3 of 24 (12.5%) with type 2, 5/31 (16.1%) with type 5. Thus, as high risk of death we included patients with CT classification types 3, 4 and 6, with a combined mortality rate of 19/45 (42.2%); and as low risk of death we included patients with CT classification types 2 and 5, with a combined mortality rate of 8/55 (14.5%). We calculated Odds Ratios and 95% confidence intervals as measures of the clinical impact of the predictor variables.

SPSS 17.0 (SPSS Inc., Chicago, IL, USA), NCSS 2000 (Kaysville, Utah) and LogXact 4.1, (Cytel Co., Cambridge, MA) were used to perform statistical analyses. All *P* values lower 0.05 were considered statistically significant

## Results

Comparisons of demographic and clinical parameters between surviving (N = 73) and non- surviving (N = 27) patients are shown in Tables 1 and 2. Non-surviving TBI patients showed lower GCS, higher age, female rate and APACHE-II score than survivors. We found statistically significant differences in CT classification between non-surviving and surviving patients. In addition, non-surviving patients showed higher serum CCCK-18 levels than survivors (P = 0.003).

**Table 1:** Comparison of computer tomography findings between non-surviving and surviving patients with traumatic brain injury.

	**Non-survivors** (n = 27)	**Survivors** (n = 73)	P value
Computer tomography classification—n (%)			0.002
· Type 1	0	0	
· Type 2	3 (11.1)	21 (28.8)	
· Type 3	5 (18.5)	13 (17.8)	
· Type 4	6 (22.2)	10 (13.7)	
· Type 5	5 (18.5)	26 (35.6)	
· Type 6	8 (29.6)	3 (4.1)	

**Table 2:** Comparison of clinical and biochemical characteristics between non-surviving and surviving patients with traumatic brain injury.

	**Non-survivors** (n = 27)	**Survivors** (n = 73)	P value
Age (years)	66 (45–76)	47 (32–67)	<0.001
APACHE-II score	26 (25–32)	19 (17–23)	<0.001
aPTT (seconds)	26 (25–31)	28 (25–32)	0.86
Bilirubin (mg/dl)	0.75 (0.53–1.05)	0.50 (0.40–0.87)	0.045
CCCK-18 (U/l)	347 (160–401)	180 (151–224)	0.003
CPP (mmHg)	60 (54–69)	68 (57–70)	0.46
Creatinine (mg/dl)	0.95 (0.70–1.10)	0.80 (0.70–0.90)	0.44
Female gender—n (%)	11 (40.7)	12 (16.4)	0.02
Fibrinogen (mg/dl)	376 (246–560)	350 (282–444)	0.32
Glasgow Coma Scale score	3 (3–6)	7 (6–8)	<0.00171
Glycemia (g/dL)	161 (142–189)	139 (120–163)	0.08
Hemoglobin (g/dL)	11.1 (9.4–12.3)	11.4 (10.4–13.0)	0.87
ICP (mmHg)	20 (12–30)	15 (14–20)	0.27
INR	1.22 (1.01–1.67)	1.03 (0.92–1.15)	0.15
ISS	25 (25–27)	25 (25–32)	0.24
Lactic acid (mmol/L)	1.90 (1.15–4.55)	1.70 (1.23–2.50)	0.16
Leukocytes *10^3^/mm^3^	18.3 (10.7–23.9)	14.7 (10.2–19.3)	0.46
PaO2 (mmHg)	141 (104–186)	151 (116–217)	0.34
PaO2/FI0_2_ ratio	190 (154–316)	336 (242–407)	0.11
PaCO2 (mmHg)	39 (35–40)	39 (36–31)	0.80
Platelets *10^3^/mm^3^	215 (139–264)	182 (143–252)	0.48
Sodium (mEq/L)	141 (135–149)	139 (138–142)	0.19
Temperature (°C)	36.0 (35.0–37.0)	37. (35.6–37.3)	0.12

Data are shown as median (percentile 25^th^-75^th)^ or number (%); APACHE II = Acute Physiology and Chronic Health Evaluation; aPTT = activated partial thromboplastin time; CCCK = caspase-cleaved cytokeratin; CPP = cerebral perfusion pressure; ICP = intracranial pressure; INR = international normalized ratio; ISS = Injury Severity Score; PaO_2_ = pressure of arterial oxygen; FIO_2_ = fraction inspired oxygen; PaCO_2_ = pressure of arterial carbon dioxide

**Fig 1:**
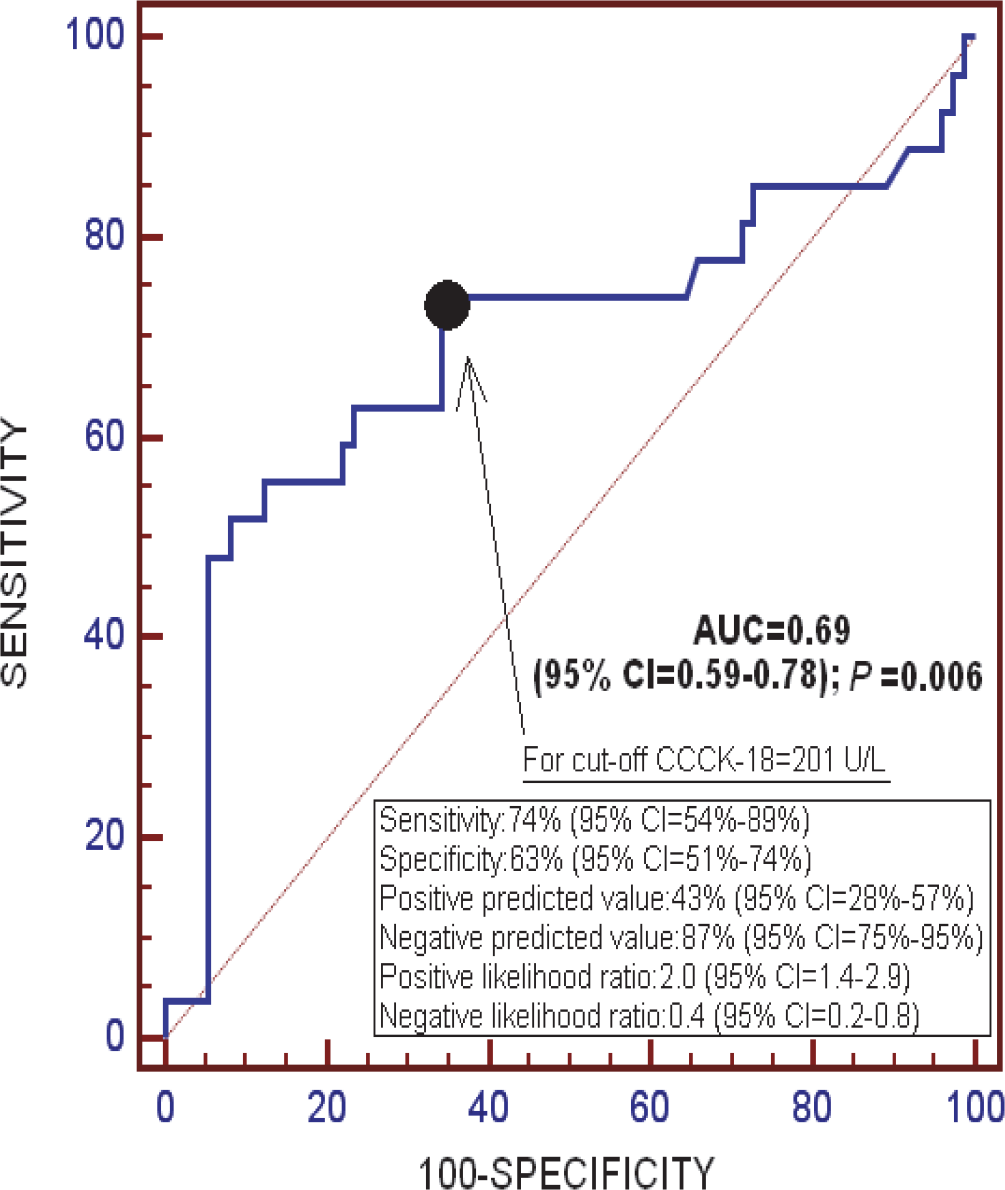
Receiver operating characteristic analysis using serum caspase-cleaved cytokeratin (CCCK)-18 levels as a predictor of mortality at 30 days.

On ROC analysis, the area under the curve (AUC) for serum CCCK-18 levels as predictor of 30-day mortality was 0.69 (95% CI = 0.59–0.78; P = 0.006) (Fig. 1).

**Fig 2:**
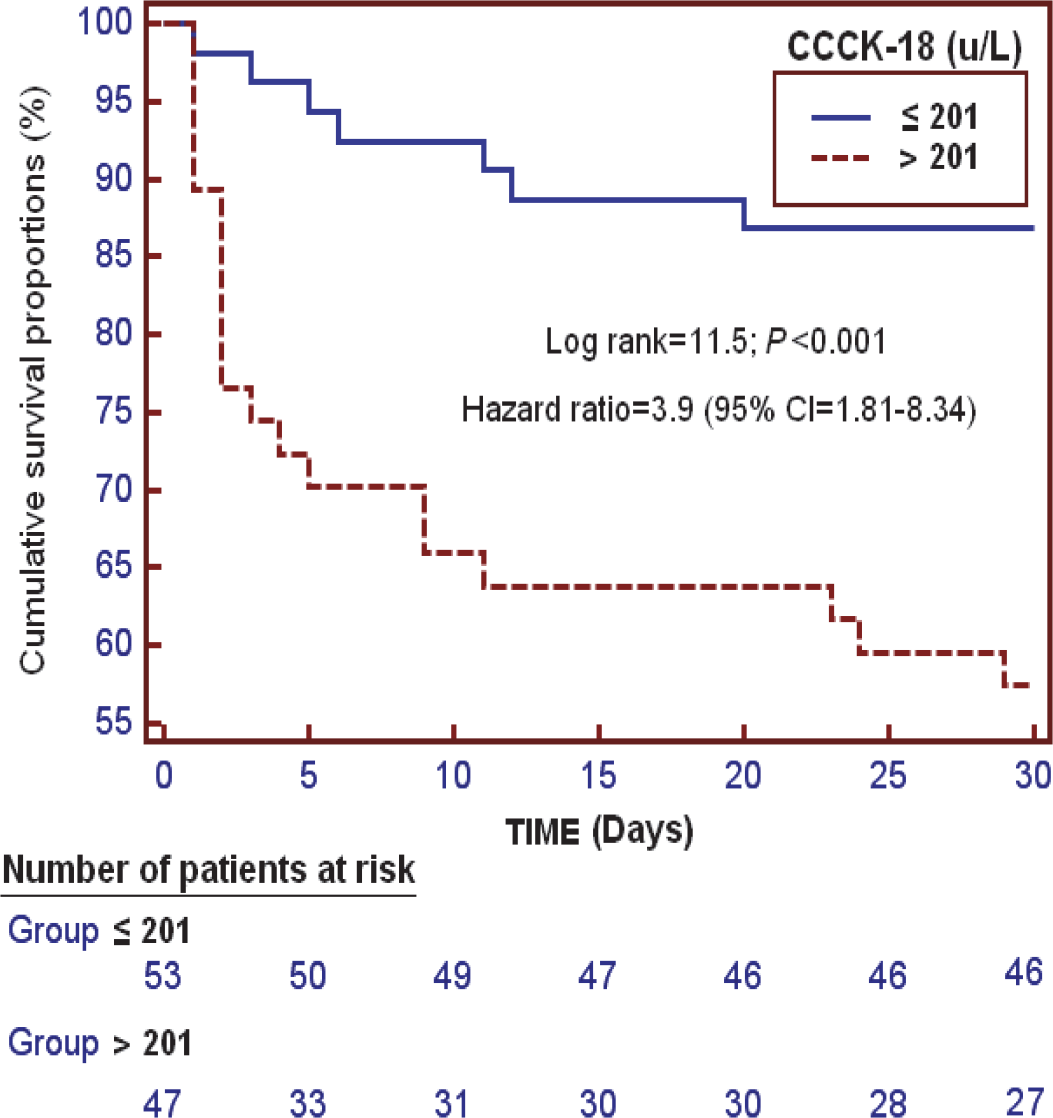
Survival curves at 30 days using serum caspase-cleaved cytokeratin (CCCK)-18 levels higher or lower than 201 u/L.

We found in survival analysis that patients with serum CCCK-18 higher than 201 u/L had a higher 30-day mortality than patients with lower levels (Hazard ratio = 3.9; 95% CI = 1.81–8.34; P<0.001) (Fig. 2).

**Fig 3:**
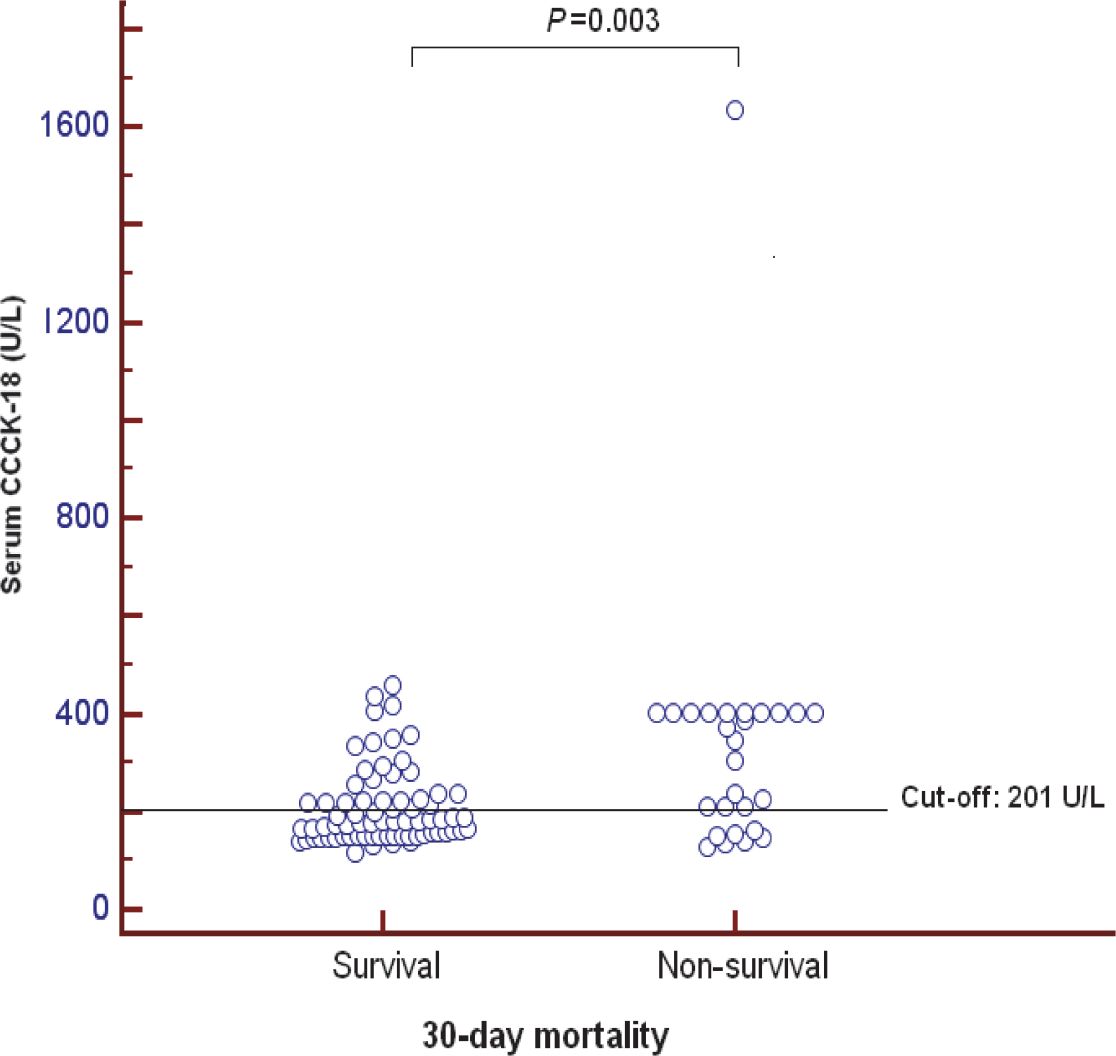
Dot-plot of serum caspase-cleaved cytokeratin (CCCK)-18 levels in 30-day surviving and non-surviving patients.

We ploted serum CCCK-18 levels in 30-day surviving and non-surviving severe TBI patients (Fig. 3).

Regression analyses showed that serum CCCK-18 levels higher than 201 u/L were associated with 30-day mortality (OR = 8.476; 95% CI = 2.087–34.434; P = 0.003) after controlling for age and GCS (Table 3). Similarly, serum CCCK-18 levels higher than 201 u/L were associated with 30-day mortality (OR = 9.789; 95% CI = 2.196–43.643; P = 0.003) after controlling for CT classification and APACHE-II (Table 3).

**Table 3:** Multiple binomial logistic regression analysis to predict 30-day mortality.

Variable	**Odds Ratio**	**95% Confidence Interval**	***P***
**First Model**			
Age	1.085	1.040–1.133	<0.001
GCS score	0.599	0.433–0.828	0.002
Serum CCCK-18 levels>than 201 u/L	8.476	2.087–34.434	0.003
**Second Model**			
Computer tomography classification (reference category: low risk of death)	6.971	1.559–31.166	0.01
APACHE-II score	1.450	1.220–1.723	<0.001
Serum CCCK-18 levels>than 201 u/L	9.789	2.196–43.643	0.003

GCS Glasgow Coma Scale; CCCK = caspase-cleaved cytokeratin; APACHE II = Acute Physiology and Chronic Health Evaluation

## Discussion

To our knowledge, no previous work has investigated the relevance of serum CCCK-18 levels as a marker of apoptosis in patients with severe TBI. The major finding our study was that serum CCCK-18 levels higher than 201 u/L were associated with a 4-fold increase in 30-day mortality. The clinical relevance of this finding is that this parameter could be used as a prognostic biomarker of early mortality in TBI patients.

The rol of apoptosis in TBI remains unclear. However, there are data in animals [3–5,29,30] and human patients suggesting that apoptosis is present in TBI [6,7]. In a study with rats subjected to mild lateral fluid-percussion brain injury were found morphologic characteristics of apoptotic cell death in tissue homogenates [3]. In another study was performed a mild-moderate controlled cortical impact injury in mice and were found apoptotic changes in brain issues [4]. In a rabbit model study of ventricular fluid impact were found apoptotic changes in brain samples [5]. In TBI rat models were found a higher expression of pro-cell death genes (such as Bax) and a lower expression of anti-cell death genes (such as bcl-2 and bcl-xL) in brain tissue after TBI [29,30]. Clark et al compared brain tissue samples removed from TBI adult patients during surgical decompression for intracranial hypertension or obtained at autopsy from non-trauma patients (controls); and the authors found increased apoptotic cells in tissue from TBI patients compared with controls [6]. Miñambres et al determined the presence of apoptotic cells in brain samples from patients with TBI and found that apoptotic rate was associated with mortality [7].

From a therapeutic perspective, modulators of apoptotic activity could be used as a new class of drugs for the treatment of TBI. In a rat model, intrathecal infusion of a caspase-3 inhibitor was reported to reduce apoptosis, contusion size and brain tissue loss, but no effect on functional outcome was observed [31].

The strengths of the present work are that it was a multicenter study (which increases the possibility of applying its findings to TBI patients in other similar intensive care units) and that the sample size was sufficiently powered to be able to report for the first time an association between serum CCCK-18 levels and 30-day mortality. However, our study also had certain limitations. First, we did not perform an analysis of serum CCCK-18 levels at different time points during follow-up to compare the evolution of this parameter between non-surviving and surviving TBI patients. Second, measuring the levels of other compounds of the apoptotic state would be desirable to better evaluate this process. Third, we have not data about apoptosis in brain tissue; thus, we have not determine whether there is an association between serum CCCK-18 levels and brain apoptosis, and what is what happens in situations of regional differences in tissue (penumbra/ contusion vs. pericontusional tissue/ normal tissue). Four, the determination of total serum CCCK-18 levels by M65 ELISA or M65 EpiDeath ELISA would have been interesting in order to quantify the leading mode of cell death (apoptosis or necrosis). Thus, additional studies are needed to confirm the results of our study.

In conclusion, the novel finding of our study was that serum CCCK-18 levels are associated with 30-day mortality and could be used as a prognostic biomarker in patients with severe TBI.
